# Long-lived monolithic micro-optics for multispectral GRIN applications

**DOI:** 10.1038/s41598-018-25481-x

**Published:** 2018-05-09

**Authors:** Antoine Lepicard, Flavie Bondu, Myungkoo Kang, Laura Sisken, Anupama Yadav, Frederic Adamietz, Vincent Rodriguez, Kathleen Richardson, Marc Dussauze

**Affiliations:** 1 Université de Bordeaux, Institut des Sciences Moléculaires, UMR 5255 CNRS, 351 Cours de la Libération, 33405 Talence Cedex, France; 2 Department of Materials Science and Engineering, College of Optics and Photonics, University of Central Florida, Orlando, FL United States

## Abstract

The potential for realizing robust, monolithic, near-surface refractive micro-optic elements with long-lived stability is demonstrated in visible and infrared transmitting glasses capable of use in dual band applications. Employing an enhanced understanding of glass chemistry and geometric control of mobile ion migration made possible with electrode patterning, flat, permanent, thermally-poled micro-optic structures have been produced and characterized. Sub-surface (t~5–10 µm) compositional and structural modification during the poling process results in formation of spatially-varying refractive index profiles, exhibiting induced Δn changes up to 5 × 10^−2^ which remain stable for >15 months. The universality of this approach applied to monolithic vis-near infrared [NIR] oxide and NIR-midwave infrared [MIR] chalcogenide glass materials is demonstrated for the first time. Element size, shape and gradient profile variation possible through pattern design and fabrication is shown to enable a variety of design options not possible using other GRIN process methodologies.

## Introduction

Micro-optics have gained significant interest in the past decade. This field of optics has led to the creation of components or devices with optical functions that often cannot be envisioned or attained with conventional optics. Components in micro-optics commonly rely on diffractive or refractive surfaces, finding applications in beam shaping, as beam splitters, diffusers, gratings, correction plates or as other components^1^. These surfaces are usually created by imprinting, etching, or other lithographic means. The decrease in components’ size enabled by micro-optical elements has allowed large and complex optical systems to be miniaturized^2^ and the development of equipment directly benefiting from their small sizes, such as microlens arrays for plenoptic photography and gigapixel microscopy with high throughput^3–5^. In military applications for instance, diffractive optical elements (DOEs) have become essential in that they contribute to the reduction of the Size, Weight and Power consumption (SWaP) of devices. Similarly, it is predicted that the use of robust, long-lived components based on gradient refractive index (GRIN) profiles which combine the optical functionality of multiple components would extend this reduction in SWaP and system cost. While GRIN fabrication strategies have examined creation of bulk optics in oxide^6–8^ and chalcogenide glasses^9,10^, CVD glass films^11^ and polymer^12–14^ materials, these efforts have primarily focused on elements where the dimension of the radial or axial refractive index gradient is over the range of a few to 10’s of millimeters, not at the micron level suitable for micro-optical applications.

Several techniques are available to create micro-optical components ranging from wet or dry etching structures into glasses, to microlithographic methods using polymers^1,15–17^. The precision of these manufacturing methods dictates the size and complexity of the micro-optical components to be created as well as the materials that can be used. Recent efforts on bulk GRIN materials have employed multi-material (layered) approaches^18^. These etch- or laminate-based approaches often rely on multi-step, time-consuming, and composition-specific protocols, even within similar material matrices (i.e., glasses or polymers). Depending on processing methodology, the resulting surface modification may exhibit short term (temporal) stability to the induced index or topographic modification under ambient conditions, fragility issues based on post-etch surface relief topography or environmentally-induced discontinuities of optical and physical properties at layer interfaces. The proposed effort employs treatment of a monolithic glass’ surface, to realize creation of near-planar micro-lens arrays which do not require such cumbersome fabrication protocols. Applicable to a range of oxide or chalcogenide glasses, the poling treatment enables design and fabrication of uniform, micron-scale lenslets compatible for use in small footprint, dual band applications.

Thermal poling has been initially developed for glass-to-metal sealing (also known as anodic bonding) in the microelectronics industry by Wallis and Pomerantz^19,20^. The principle consists of placing a glass sample between two electrodes at elevated temperature (typically below the glass transition temperature) and the application of a dc electric field across the sample to impart field-assisted migration of specific mobile ions. In the 1990s, poling gained interest for nonlinear optics when it was observed that a stable space charge could be induced below the anodic surface resulting in an induced second order nonlinear optical property not present in isotropic glass^21–23^. Further studies demonstrated that this subsurface layer underwent large structural and compositional changes in ionic glasses^24–27^. Such changes were also shown to yield modification of other glass properties, such as mechanical and optical properties^28,29^. More recently, it has been demonstrated that micro-structuring of the glass was possible using patterned electrodes. This methodology was applied to various glass systems^30–35^ and used for local changes in glass surface reactivity control^36,37^, micro-structuring of nonlinear optical properties^34^, and the fabrication of waveguides and diffractive gratings^38–41^. Despite these examples, no systematic effort to exploit the geometrical control of the ion migration mechanism to enable formation of deliberate spatial profiles of the mobile ion depletion resulting in periodic structures (i.e., arrays) where optical function can be manipulated through specific glass material selection and poling condition tailoring has been reported. An understanding of these inter-relationships including mechanistic details of the induced field behavior in chalcogenide glasses^42^ and the parallel impact on non-optical properties^43^, has been carried out, and the aspects related to the formation of dual band micro-GRIN lens arrays, are reported here.

In this article, we demonstrate that this technique can be applied on a variety of glasses to create micro (μ)-GRIN optics. Specifically, we present the applicability of thermal poling as a flexible method to create linear optical property modification across multiple glass types. The universality of this approach applied to vis-NIR (oxide) and NIR-MIR (chalcogenide) materials is demonstrated for the first time and we show how element size, shape and gradient profile variation is possible through pattern design. This flexibility enables a variety of design options not previously realized using other GRIN process methodologies and is shown in illustrative examples of dual band microlens arrays.

Glasses to be structured vary in compositions, but all contain a certain amount of mobile species, most frequently monovalent alkali ions. The method has been successfully applied to oxide glasses (soda-lime, niobium borophosphate, borosilicate) and non-oxide (chalcogenide) glasses. To highlight the mechanisms behind the μ-GRIN formation, we first focus on the study of a chalcogenide glass (ChG) of the composition Ge_22.5_Sb_10_S_67.5_ doped with 3 mol% sodium sulfide, where the glass composition can be re-written as Ge_31.1_Sb_23.2_S_43._Na_2.6_ (in weight percent). The preparation procedure of this glass can be found elsewhere^43^. These non-oxide glasses are especially interesting as they have a high refractive index, are transparent in the near- and mid-IR region and have easily tunable properties that can be varied with composition.

## Results and Discussion

**Figure 1:**
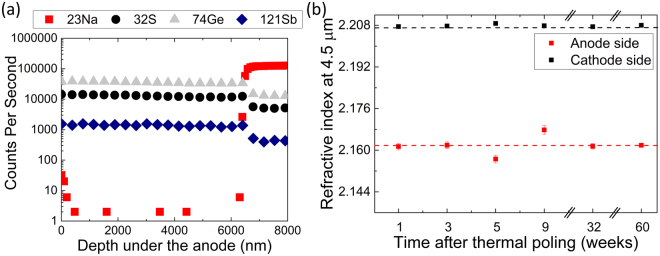
Secondary Ion Mass Spectrometry (SIMS) profile recorded on a glass doped with 3 mol% of Na_2_S after thermal poling (**a**) and refractive index values measured at λ = 4.5 µm on the cathode and anode sides of a poled sample doped with 3 mol% of Na_2_S (**b**). Error of measurements are within the data point and the dashed line is presented as a guide to the eye.

To better grasp the mechanisms behind the formation of the gradient of index, we first poled a glass sample using a homogeneous, planar electrode at the anode. Secondary Ion Mass Spectroscopy (SIMS) was used to measure the glass’ various constituents while a Focused Ion Beam (FIB) milled through the first microns beneath the anodic surface. A representative depth profile recorded after thermal poling is shown on Fig. 1(a). Evidence of compositional variations due to the treatment is shown as a sodium depletion layer 6.5 μm in depth is formed. The other glass constituents remain unchanged (the small decrease in signal once sodium returns to its starting level in the glass’ bulk is an artifact due to charging of the sample). The resulting impact of this compositional variation on the glass’ refractive index was then investigated using a modified IR-Metricon prism coupler at λ = 4.5 μm. This system possesses a measurement resolution of +/−0.0005^44^. To evaluate the impact of sodium concentration on the refractive index value, an alkali-free glass sample was used as a reference and its index was measured to be 2.181. The refractive index of the doped glass exhibits an index of 2.207, showing that addition of sodium to the parent glass leads to an increase of refractive index, likely due to a modification in local density. After thermal poling, the refractive index was re-measured on both sides of the sample. On the poled sample, the anode side corresponds to the poled (sodium-depleted) area and the cathode side to the unpoled area (SIMS measurements confirmed that no compositional changes take place on the cathode side). Figure 1(b) shows the refractive index values measured on both sides of the samples as a function of time. One can see a decrease in refractive index of −0.045 after thermal poling on the anode side while the cathode side’s refractive index remains unchanged. The error bars (where shown) represent the index variation as measured under multiple locations indicating the small but measurable compositional variation in the depleted layer. It is worth noting that the refractive index in the poled area is even lower than that of the parent glass free of sodium. From this observation, we suggest that the change in refractive index is not simply related to the sodium migration but rather to a combination of effects. In addition to the local compositional change, structural reorganizational changes due to the Na^+^ departure are likely taking place to ensure charge neutrality in the post-poled structural arrangement. These changes would impact local density and molar volume variation, both attributes that modify the polarizability defining the local refractive index and therefore further decreasing the refractive index. One could postulate that the *local* relaxation behavior within these two varying compositions (at the anode or cathode regions) now co-existing within the same bulk sample, might be different due to their expected variation in glass transition temperatures due to differences in Na^+^ content. Lastly and importantly for applications exploiting this effect, this study demonstrates that the refractive index change exhibits excellent temporal stability as subsequent measurements showed no variation with time. Index measurements made more than a year after poling remain unchanged due to compositional tailoring of the parent glass to stabilize the post-poled glass network^32^. The slight variations observed on two points on the anode side at weeks 5 and 9 are off the trend (dashed) line shown, and could be associated with local inhomogeneities (in Na^+^ content or in post-poled glass network structure) within the measurement volume sampled over the multiple measurements. Alternatively, the offset in these two points could be attributed to operator-induced measurement errors. As can be seen this offset was only in these interim dates and when repeated several weeks later, values returned to the trend line shown. As samples were stored at room temperature during this time, we confirm the lack of relaxation effects under ambient laboratory conditions, a critical aspect for use in future applications.

**Figure 2:**
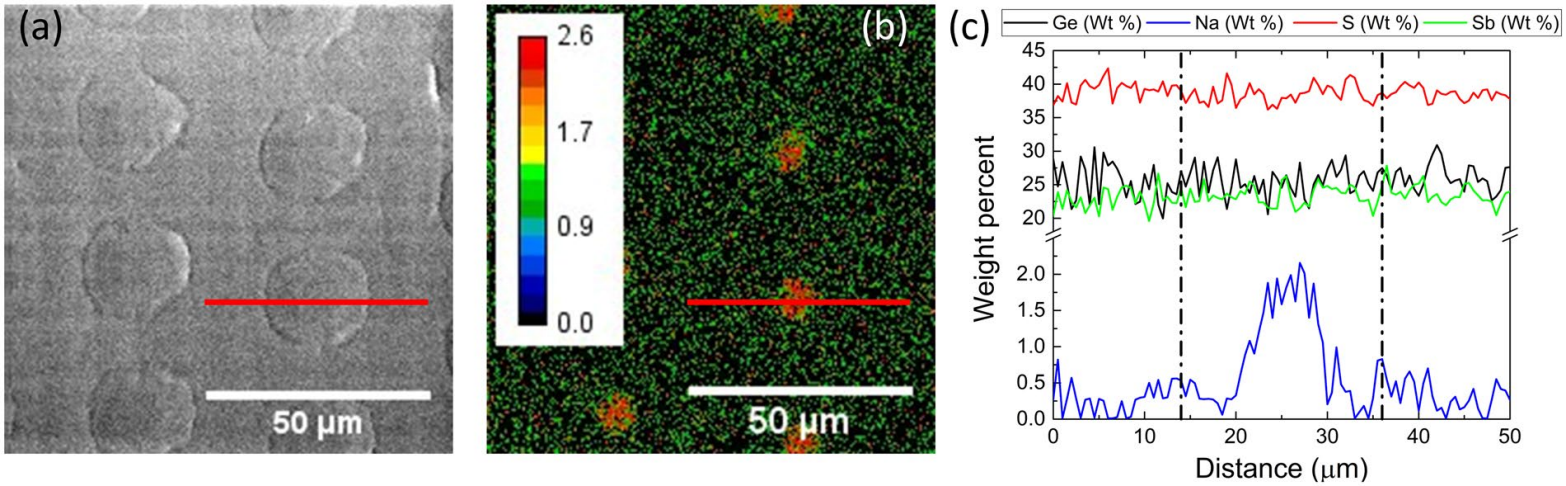
BSE image of patterns imprinted on the glass surface (**a**), map of the Na distribution in wt% measured by WDS (**b**), and elemental profile (wt %) of Ge, S, Sb and Na across the shown (in b) red line, vertical dash lines represent the edges of the pattern (**c**).

Now that we have observed macroscopic changes in refractive index following thermal poling, we aim to tailor these changes to create micro-structured optics. The goal is to induce gradual compositional changes to create gradients of refractive index at the micrometer scale in the sub-surface region thereby creating GRIN structures without imparting surface relief (topography) to the planar surface. Using procedures described in^24^, a 100-nm thick indium tin oxide (ITO) layer deposited on a microscope slide layer was fabricated to form a structured electrode by laser ablation. The now patterned ITO layer is comprised of conductive areas (where ITO remains) and non-conductive areas (where ITO has been fully ablated and removed, leaving a void). Following this step, the patterned electrode can be used as a stamp during thermal poling. No sign of glass/electrode interaction (corrosion) was observed in the poling of either chalcogenide or oxide glasses. The specific configuration of the anode includes a structured conductive layer sandwiched between two dielectrics, (i) the glass to be poled and (ii) the microscope slide substrate of the anode, which favors the in-plane component of the induced electric field during poling^34^. The applied electric field is therefore effective in areas that are not in contact with the conductive coating with strong in-plane components. Excellent transfer of the electrode pattern to the surface was observed with minimal surface relief changes (RMS < 10 nm). The minimal surface change may be linked to mechanical stresses due to the departure of mobile modifiers from the near surface region. Regions now void of modifiers possess differences in local molar volume and can exhibit partial volume relaxation at the processing temperatures used. The change in volume is maximum in areas of contact between the electrode and the glass surface. While not fully optimized in the present study, it is possible to further optimized the surface relief change by changing the poling conditions and alkali content in the base glass, as proposed by Brunkov *et al*.^45^, and noted in other alkali doping levels examined in the present system^43^. It is worth pointing out that in some oxide glasses the surface relief change has been measured to be as high as hundreds of nanometers in compositions with a high alkali and alkali-earth content (20 weight %)^33^. Micro-probe analysis was then performed using Wavelength Dispersive Spectrometry (WDS) and Energy Dispersive X-ray spectrometry (EDX) in a scanning electron microscope (SEM) to measure the distribution of the various elements, Ge, Sb and Na in the patterned areas. The back-scattered electron (BSE) image was centered on six, 25-µm diameter circles imprinted on the glass surface, Fig. 2(a), and compositional maps were recorded. No variation in the glass’ network forming species (Ge, Sb and S) were observed, remaining homogeneously distributed within the studied area, as shown in the pattern profile in Fig. 2(c). However, changes in the radial distribution of the sodium in the surface layer were measured with a strong decrease in the sodium levels outside the patterned areas whereas a Na-rich region remained present in the areas void of electrode material (the center), Fig. 2(b). Note that the composition in the center of the pattern is close to that of the glass before poling while the composition outside the patterned area is close to the one of the base glass without Na_2_S. The reason behind this compositional variation is easily explained as related to the structuring of the electrode which impose both electric field strength and geometry. As stated earlier, the specific anodic configuration used in this example favors an in-plane component of the outside electric field. The sodium migration hence follows the electric field lines and the mobile species are pushed deeper inside the monolithic glass sample in regions in contact with the conductive ITO coating. However, in the structured, ITO-free areas, the gradual decay of the surface potential leads to a gradient of sodium migration where minimal migration takes place in the center of the patterned areas. This hypothesis is confirmed when plotting a cross-section line of the sodium content through a circle and comparing it to the BSE image, Fig. 2(c). It is observed that the limit between the depleted and undepleted area is not sharp but follows a gaussian-shaped distribution, consistent with field-induced ion mobility through diffusion. A spatially varying gradient of composition directly related to the electrode pattern shape, is therefore induced in the near-surface region. While each of the other glass species show no variation in their quantities as a function of position, we would expect that structural re-organization has occurred between these network participants to compensate for both the charge and molar volume changes resulting during poling. While shown for a circle in this example, other feature geometries (simple gratings or other shapes) can readily be created both in the electrode and imprinted on the glass.

**Figure 3:**
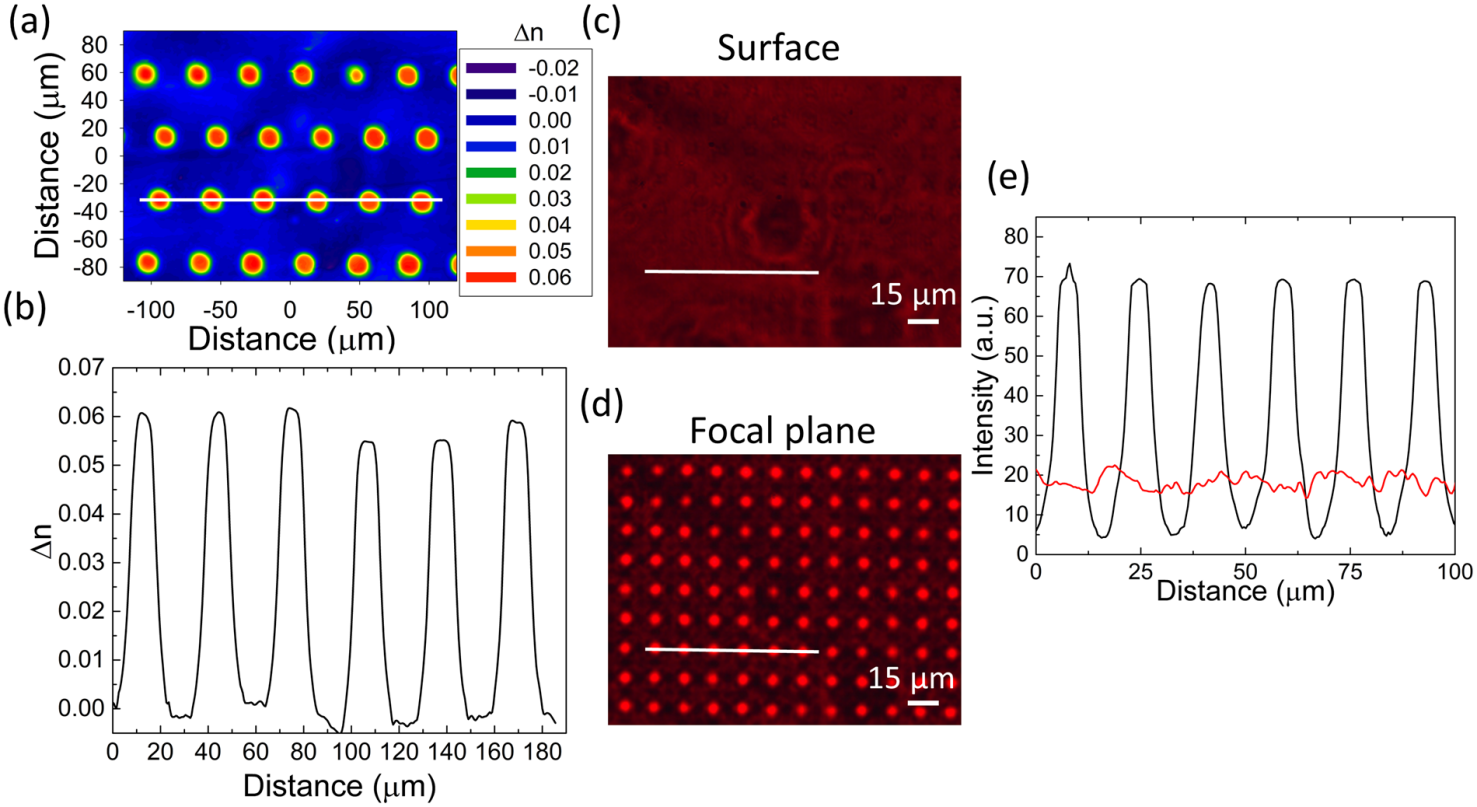
PHASICS map of a two-dimensional array of circles imprinted on a ChG glass with index changes (Δn) shown as color changes (**a**), Δn linescan profile measured on the PHASICS map (**b**), light intensity at the surface of the sample (**c**), light intensity at the focal plane of the microlens array (**d**), comparison of intensity profiles recorded at the surface (red) and at the focal plane (black) (**e**).

Such compositional and structural changes lead to refractive index variations. To obtain an image of the refractive variation across these patterns, a PHASICS SID4-bio apparatus allows measurement of the phase image with high spatial resolution. The camera measures the change in optical thickness within the sample which is impacted by any variation in refractive index. As noted, there was negligible (RMS < 10 nm) surface modification of the poled sample. The refractive index variations within individual lenslets or across the array of lenses can then be determined if the thickness of the poled area is known. Figure 3(a) shows a PHASICS map of a similar area within this poled sample measured at λ = 750 nm. Note, that the non-oxide glass used here remains transmissive in this region enabling measurement. Here, the thickness of the poled near-surface area was approximated to be equal to that found by SIMS measurements. A refractive index variation of Δn ∼ 0.06 was measured, Fig. 3(b), which is of the same order of magnitude found in direct refractometer measurements at λ = 4.5 µm. This demonstrates that the absorption edge (~600 nm) does not impair the index mapping characterization at 750 nm. In the present case, we observe a Gaussian like profile clearly defining the formation of a gradient of refractive index (GRIN) profile. This result is correlated to the compositional variations observed previously. The possibility to create GRIN micro-structures in a near surface region of an optical glass enables direct application to the realization of optical devices. Of special interest is the formation of planar microlens arrays using thermal poling. GRIN materials are especially useful in compact systems with high power for aberration correction. To evaluate the potential of these features as microlenses, we examined the sample in a confocal microscope in transmission mode with a collimated monochromatic incoherent source. The microscope objective was first focused on the surface of the glass (z = 0, Fig. 3(c)) and slowly defocused away from the sample until the matrix illuminated (z = focal of microlenses, Fig. 3(d)) thereby directly retrieving a first approximation of the microlenses’ focal lengths, here equal to 125 µm. Two profiles were extracted from these images and are shown on Fig. 3(e). The intensity profile at the surface is shown in red while the intensity profile at the focal plane is shown in black. At the focal plane, the light intensity is measured to be four times higher than at the surface, Fig. 3(e).

**Figure 4:**
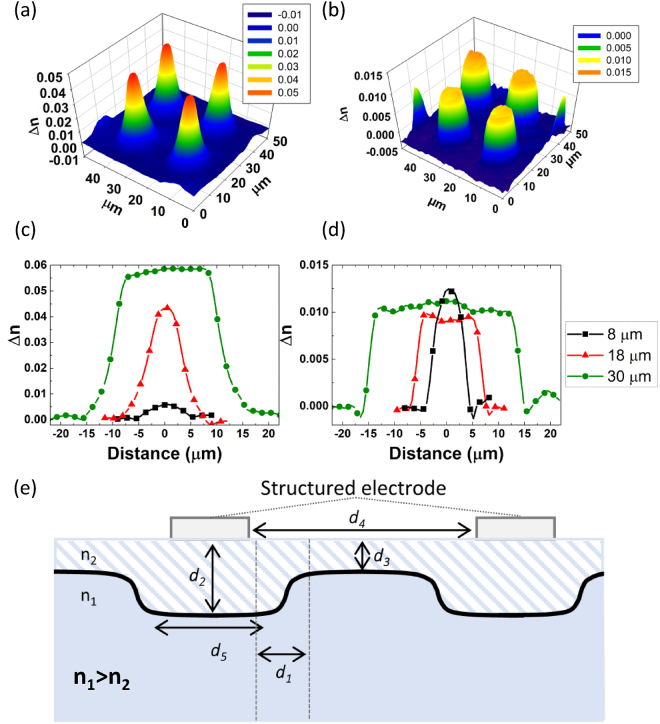
Phasics map at λ = 750 μm of 18 µm spots imprinted on a chalcogenide (**a**) and soda lime glass (**b**) subjected to identical poling voltages; Δn profiles measured across circles 8, 18 and 30 µm wide on a chalcogenide (**c**) and soda lime glass (**d**); principle parameters controlling the gradient of index geometry in the glass after thermal poling (**e**).

To evaluate the universality of the process on a wider variety of glasses, electrodes with different features (circles, squares, honey comb) were prepared and employed on chalcogenides and oxide glasses (niobium borophosphate, soda lime silicate, germano-gallate). All samples were poled at temperatures below their glass transition temperature and subjected to an electric field of approximately 1 kV. To highlight the role of the glass composition, a chalcogenide glass and a soda-lime glass (SLG) were poled with the *same* electrode (consisting of round spots, 18 μm in diameter) and the resulting PHASICS maps were compared as shown in Fig. 4(a) and (b). For a similar feature size, one can compare the shape of the gradient and its variation in the two glasses. Firstly, the magnitude of Δn is higher in the chalcogenide, 0.06 versus 0.015 in the SLG. Employing the same electrode, the soda lime glass’ index profile exhibits a flat top while the ChG has a Gaussian-like profile of variation, highlighting the two glasses matrices’ composition-specific impact on alkali mobility under the applied field. Commercial SLG (composition: SiO_2_ 72.2%, Na_2_O 14.3%, CaO 6.4%, MgO 4.35%, K_2_O 1.2%, Al_2_O_3_ 1.2%, SO_3_ 0.3% in weight percent) contains numerous constituents including other modifiers – either mono- or divalent-oxides which can be impacted (mobilized) by the applied field which have been previously observed and modelized^28,46^. Conversely, the custom ChG composition contains only Na^+^ added as the mobile species. Hence, the species mobility as well as the molar volumes of the two glasses are quite different and the resulting behavior is reflective of those differences. The resulting alkali and index profile variation in the post-poled glass defines the observed focal lengths of the micro-lenslets which were measured and found to be 65 and 110 µm, respectively. These results can be directly correlated to both the magnitude of the refractive index change and to the geometry of the gradient imparted by the electrode pattern and the poling conditions employed. As expected, as the Δn increases so does the optical power of the resulting lens, thus resulting in a shorter focal length. In an attempt to tune the optical power of the resulting optic and to highlight the role of the glass’ matrix on the final microlenses’ properties, two additional spots, 8 and 30 µm in diameter were inscribed on the same glasses. The change in shape of the gradient on both glasses are shown on Fig. 4(c) and (d) and can be illustrated by the diagram shown on Fig. 4(e). As depicted, a thin layer depleted of sodium ions is formed beneath the anode with a refractive index n_2_ lower than that of the bulk glass. The thickness and shape of this modified layer varies depending on the structuring of the electrode which dictates the field distribution and gives an effective refractive index that varies gradually. In both glass systems, the highest Δn is found at the widest point which also presents a flat top. We observe that the refractive index reaches its maximum value at a distance *d*_1_ (see Fig. 4(e)), specific to the glass composition. By varying the diameter *d*_4_ of the pattern, it is possible to control the shape of the induced gradient, as well as the lens’ diameter. For *d*_1_ < *d*_4_/2 no depletion occurs in the center of the pattern and a flat top starts to appear (see point 30 µm wide in Fig. 4(c) and points 18 and 30 µm wide in Fig. 4(d)). The magnitude of the induced Δn is maximum and d_3_ is null. To obtain a Gaussian shape and a maximum Δn, the diameter is set to twice *d*_1_ as observed for the 18 µm-points (ChG) and 8 µm-points (SLG) on Fig. 4(c) and (d) respectively. Finally, for *d*_1_ > *d*_4_/2, the depletion also takes place in the center of the feature, which therefore decreases the magnitude of the induced Δn, as seen on 8 µm-points Fig. 4(c). In addition, the distance *d*_3_ starts to increase. These different examples demonstrate that it is possible to precisely control the GRIN formation within a specific glass type by controlling the distance *d*_1_. This distance is specific to a glass composition and can be further tuned by changing the various poling parameters. Additionally, while applied to circular geometries (lenslets), the methodology described here can similarly be extended to diffractive elements such as gratings.

## Conclusions

In summary, this article demonstrates for the first time the magnitude, and spatially varying impact of poling-induced composition and index modification to realize micro-lens arrays. Specific examples report the magnitude of the refractive index changes seen in representative oxide and chalcogenide glasses to be large (up to Δn ~ 5 × 10^−2^) with no post-poling degradation to optical quality making the candidate components suitable for use in dual band optical systems. Micro-optical lens arrays realized by inducing a gradient refractive index (GRIN) profile have been designed, fabricated and characterized in both oxide and chalcogenide glasses containing mobile cations. Post-poled index modification to the glasses are shown to exhibit outstanding stability (>15 months) not previously realized in such structures with the induced index profile created showing negligible changes to surface topography. We have illustrated the relationship between electrode pattern and the resulting microlens’ power, though further studies are necessary to more fully control the shape and magnitude of the mobile ion migration responsible for the induced change. These advances will enable formation of target gradient designs encompassing specific slope and magnitude of a desired refractive index profile. While this work presents proof of our concept illustrating the fabrication of two dimensional micro-lens arrays, the same protocol can be widely extended to other diffractive and refractive geometries including waveguides^28,39^, and diffractive gratings^41,47^ using spatially-specific electrode design, poling condition optimization and material compositional tailoring. We envision this method will emerge as a means to tailor the near-surface optical properties in bulk and thin film glasses enabling the creation of diverse, multi-spectral optical devices such as diffusers, beam splitters, and corrector plates.

## Methods

### Glass synthesis

The chalcogenide glass was prepared from high purity elemental Ge, Sb and S (Alfa Aesar, 99.999% purity) in 30 grams batches. Sodium was incorporated to the matrix using sodium sulfide (purity unspecified). Raw materials were weighed out under nitrogen in a glove box and inserted in a quartz ampule. A closed connector was placed at the end of the tube while in the glove box. The batch was then subjected to a mild vacuum for 30 minutes and then sealed with an oxygen-methane torch. The tube was placed in a rocking furnace (Barnstead Thermolyne 21100) and heated to the homogenization temperature, 850 °C, with a heating rate of 1 °C/min. The ampule stayed at the homogenization temperature overnight and the temperature was lowered to 750 °C before quenching the tube with forced air. The ampule was then place in a furnace (Barnstead Thermolyne 48000) and annealed at Tg-40 °C. The glass transition temperature of the glass is 260 °C, and is discussed in more detail, along with other physical properties, in^43^. After annealing, the glass rod was cut and polished in 1-mm thick plates.

The soda lime glass slides used during the experiments were commercial Menzel Gläser slides (T_g_ = 545 °C), with the following composition (given by the manufacturer) in weight percent: SiO_2_ 72.2%, Na_2_O 14.3%, CaO 6.4%, MgO 4.35%, K_2_O 1.2%, Al_2_O_3_ 1.2%, SO_3_ 0.3%.

### Thermal Poling

In all experiments reported here, thermal poling was performed under nitrogen atmosphere. Glass samples were heated from room temperature to the poling temperature of 210 °C for chalcogenide and 300 °C for the soda lime glass at a rate of 20 °C/min. After 10 minutes at that temperature, an electric field of 1 kV was applied for 30 minutes. The applied dc bias was removed once the sample had return to room temperature which took approximately 20 minutes.

### Refractive index measurement

A modified Metricon prism coupler (model 2010 M) was used employing a germanium prism to measure the refractive index at 4.5 µm. Each measurement was repeated ten times and averaged and a reference sample was measured prior to every measurement.

### Secondary Ion Mass Spectroscopy (SIMS)

SIMS measurements were performed at the Materials Characterization Facility at UCF by Mikhail Klimov on a PHI Adept 1010 Dynamic SIMS System.

### Microprobe analysis

All microprobe analyses were done at Placamat by Michel Lahaye on a Cameca SX 100 using wavelength dispersion spectroscopy (WDS) for the analysis of sodium, germanium and sulfur and energy dispersive spectroscopy (EDS) for antimony.

### Phase imaging

A PHASICS SID4-bio camera was used to measure the phase change in the poled area after thermal poling. The camera was coupled to a microscope equipped with a 50x objective and a band pass filter centered at 750 nm was used during the measurements.
